# Chatting: Family Carers’ Perspectives on Receiving Support from Dementia Crisis Teams

**DOI:** 10.3390/healthcare12111122

**Published:** 2024-05-30

**Authors:** Marcus Redley, Fiona Poland, Juanita Hoe, Tom Dening, Miriam Stanyon, Jen Yates, Amy Streater, Dons Coleston-Shields, Martin Orrell

**Affiliations:** 1 Department of Psychology, Anglia Ruskin University, Cambridge CB11PT, UK; 2 School of Health Sciences, University of East Anglia, Norwich NR4 7TJ, UK; 3 School of Biomedical Sciences, University of West London, London W5 5RF, UK; 4 Institute of Mental Health, University of Nottingham, Nottingham NG7 2TU, UK; 5 Research and Development, North East London NHS Foundation Trust, Ilford IG2 7SR, UK

**Keywords:** dementia, family carers, community care, carer experiences, qualitative interviews

## Abstract

Family caregivers are vital to enabling people with dementia to live longer in their own homes. For these caregivers, chatting with clinicians—being listened to empathetically and receiving reassurance—can be seen as not incidental but important to supporting them. This paper considers and identifies the significance of this relational work for family carers by re-examining data originally collected to document caregivers’ perspectives on quality in crisis response teams. This reveals that chatting, for family caregivers, comprises three related features: (i) that family caregivers by responding to a person’s changing and sometimes challenging needs and behaviors inhabit a precarious equilibrium; (ii) that caregivers greatly appreciate ‘chatting’ with visiting clinicians; and (iii) that while caregivers appreciate these chats, they can be highly critical of the institutionalized character of a crisis response team’s involvement with them.

## 1. Introduction

In the UK, policies for the care and treatment of people living with dementia are geared towards reducing inpatient admissions by supporting people to live in their own homes for as long as possible [1,2]. One element of this policy is the development of multidisciplinary community-based crisis response teams [3]. While initially conceived for working-aged people with a functional mental illness, these teams have evolved to include people living with dementia. There is, however, no blueprint as to how these services should be organized or managed. That responsibility lies with the 191 local commissioning groups and 60 mental health trusts comprising the National Health Service. Nonetheless, these services are conceived and developed as only offering a short-term time-limited service up to the point where the crisis that prompted their involvement has passed. The work of these teams can be conceptualized as comprising two activities: (i) clinical and (ii) relational [4]. The clinical activities focus on assessing the mental and physical health of persons living with dementia; reviewing medications; arranging clinical tests; and advising on managing challenging behaviors. Clinical work always and necessarily involves clinicians in a substantial amount of relational work with caregivers: listening to and discussing caregivers’ concerns and worries; offering reassurance; and instilling in caregivers the belief that they can continue caring for their spouse or relative. This relational work has often been overlooked or downplayed as not being ‘proper work’, especially in caregiving where such work is intrinsic. When relational work is discussed, it is in terms of its significance for clinicians’ practice [5] and its impact on people living with dementia [6]. We, however, aim to further its recognition and understanding by focusing on and characterizing relational work as experienced by family caregivers. With these objectives in mind, we re-examined an existing data set in which family caregivers spoke of their experiences of being supported by crisis team members.

## 2. Method

The data we re-examined came from interviews with family caregivers (Table 1). These interviews were undertaken as part of an initiative to develop quality indicators for crisis response teams and were part of a larger study, AQUEDUCT, designed to improve crisis management for older people with dementia [7]. The participants were drawn from five NHS community mental health trusts from across England, which were selected to include urban and rural settings. Consent to be interviewed and to participate in the wider study was sought by members of the participating crisis support teams. Family caregivers were provided with an information sheet describing the research by a team member and given up to three days to decide if they wished to participate or not. Out of necessity, this re-examination is impressionistic [8]; caregivers were not explicitly asked about relations with practitioners, although they did occasionally and fleetingly reveal something about what it is like to be a caregiver and to ‘chat’—the acme of relation work—with crisis team members. ‘Chatting’ is an established means for collecting clinically important information and a foundation for therapeutic relationships [9]. Yet it was the emotional texture of these family caregivers’ accounts of supporting a family member living with dementia and chatting with practitioners that struck the first author. This sensual dynamic to the interview data became the rationale for their re-examination.

Our rereading of data took participants’ views at face value [10], and no attempt was made to explore how respondents rhetorically constructed their answers [11] or how those answers might have been influenced by the interaction between interviewer and interviewee [12]. Rather, in what was a two-stage process, participants’ answers to questions about the support they received were first summarized question by question, allowing a degree of immersion in the data. Second, these summaries became the basis for identifying new and emergent themes [13], themes that were developed and refined in analytic dialogue [14] between the first two authors (MR and FP). These emergent themes [15] then became the basis for a recoding of the data. The themes we identified were (1) that to be a family caregiver is to live in a state of precarious equilibrium; (2) as indicated above, that chatting with practitioners is a significant feature of a family caregiver’s experience of receiving support from a crisis team; and (3) that while caregivers appreciate a chat with practitioners in the crisis team, they could nonetheless express views highly critical of some aspects of crisis team members’ relational work. The process of summarizing and coding the data was supported by Microsoft Word, rather than specialist software designed to code qualitative data. In describing these findings, we followed the COREQ guidelines for reporting qualitative research [16]. Pseudonyms are used throughout.

**Table 1:** Best Practice Data Re-examined.

Re-examination of Research Data	AQUEDUCT: *A*chieving *QU*ality and *E*ffectiveness in *D*ementia care *U*sing *C*risis *T*eams
Type of Data	Semistructured interviews with family carers; audio recorded and transcribed
Eligibility Criteria	A family carer of a person living with dementia who had been seen by the crisis service in the past sixth months
Interviewers	Two female postdoctoral researchers with degrees in psychology
Site and Duration of Interviews	Conducted in the respondents’ own homes, with an average duration 40 min
Date of Data Collection	September to December 2019
Respondents	A total of 10 persons: 4 wives caring for husbands; 4 husbands caring for wives; and 2 daughters caring for mothers
Interview Questions	1. What brought you into contact with (team)? 2. What happened next? 3. How did the team talk to you about ending care of the person you care for? 4. Was there anything about your involvement with the team that you thought was really positive? 5. Was there anything about your involvement with the team that you thought was less positive? 6. If you were a person with dementia experiencing a crisis, how would you like people to treat you? 7. If you were in control of a crisis team, what changes would you make? 8. Further comments
Ethical Approval	Ethical approval was obtained from the NHS Health Research Authority, ref: 16/WM/0273


Precarious EquilibriumQuote 1 Mr Trinity: They [clinicians] talk about a red mood and a green mood and you have times when you get, as a suffer-er [of dementia], get really depressed and fed up […] and the job for a carer is to bring them back into the green area Quote 2 Mr Newman: And I thought that she had gone, and it affected me to such that […] and I was really, I panicked in my subconscious mind, but I actually thought that she had gone. It smashed me to smithereens, tore me to pieces. 
The Value of ChattingQuote 3 Mr Ford: She just chatted to us to start with and then she asked questions and then she was obviously mentally re-cording how June reacted and then she asked me specific questions. Quote 4 Mrs Brown: He was pleased, do you know what I mean, his other hand came over, but I must say I don’t know whether he understood. I was a bit tearful, and again I had to go outside because it’s sad really, I am gonna be tearful now. Quote 5 Mr Philips: She can’t say anything that requires reasoning or opinions. It’s difficult to have a conversation you know, we do sit down, and we’ll talk about the old times a bit, but whereas with normal people you’d say what do you think of Brexit, well I can’t say that to Marianne because she has no opinion on it really. Criticism of Perceived Institutional FailingsQuote 6 Ms Henderson: I know they’re busy and obviously there’s a lot of crises, and this paperwork is missed a lot, but just something, say a complementary call or something like that. Quote 7 Mr Newman: The system, there’s something not, other people might find it fine, I don’t know. They might have got the knack of telephone calling or whatever, I don’t know […] but you see they’re only nine till five, five days a week. Quote 8 Mrs Leyland: It was as if they [members of the crisis intervention team] think dementia is going to go away, well you know, we have settled that problem, goodbye kind of thing. But of course, dementia is not going to go away, is it? I felt that was a bit of their attitude, oh everything is fine now, off we go kind of thing, but it is never going to be, is it.


## 3. Results

### 3.1. Precarious Equilibrium

The demands falling on carers were characterized in their accounts as sitting at an intersection between the needs and behaviors of the person they are caring for; their own strengths and frailties; and the support they can draw from their community. Spouse-carers’ descriptions of their partner often cited moods, memory loss, delusions, and paranoia, while reports of challenging behavior encompassed financial profligacy, suicidal threats, and physical violence. Mr Trinity depicts these types of demands in the context of describing his sense of responsibility for his wife’s moods (Quote 1). However, physical frailties apart from incontinence and the need for support with dressing and making cups of tea were less prominent in carers’ accounts. Similarly, carers rarely mentioned their own physical health, an exception being a carer who mentioned her acute heart condition. The support carers could draw from their community varied considerably. Some made use of local services, like dementia cafes, while others described the involvement of family members. Mr Cavendish, despite having family living close, described himself as a ‘caged bird’, only able to leave his wife for an hour and a half off each day. There were other carers whose support seemed to come entirely from the crisis team. Interestingly, carers rarely speculated on the future trajectory of their responsibilities. They seldom spoke of death and long-term admission to hospital as possibilities. When Mrs Catherine mused over the possibility of being rushed to hospital on account of her heart condition and being unable to leave her husband alone—as had happened on a previous occasion—she observed that she would have to inform the ambulance crew she was a ‘carer’. Although Mr Newman describes the emotional impact of a waking dream in which he discovers his wife slumped against the bathroom door (Quote 2), he does not follow this up with an account of what life might be like without his wife. In the absence of any articulated and interactive consideration of how their caring responsibilities might plausibly end, these family caregivers effectively live in a perpetual present. Furthermore, since caring is vulnerable to perturbations in the needs and behaviors of the person being cared for, the carer’s circumstances, and the support that carers can draw from the community, caregivers continuously have to cope with contingencies. For caregivers, this state of precarious equilibrium, rather than simply the clinical features of the person living with dementia, is the raison d’etre for crisis teams. While the socioclinical complexities of caregiving are recognized [16], this is less so for its emotional complexities.

### 3.2. Chatting

Chatting, as Mr Ford observes (Quote 3), has clinical importance, but it might also, as Mrs Leyland suggests, be a way of ‘unloading’ problems when you ‘cannot involve family too much’. However, there is more to chatting. The way clinicians speak to carers, and particularly patients, has significance in delivering support that caregivers perceive as supportive. Mrs Hillman reports that crisis team members spoke ‘beautifully’ to her husband, while Mrs Sunbeam prized the doctor for ‘not leaving mum out’, saying, ‘he’d speak to me, but it wasn’t like he was speaking about her while she was there’. The emotional significance of these courtesies, a pervasive feature of these data, should not be underestimated. Mrs Brown describes being moved to tears when her husband stood up to shake the hand of a visiting crisis team member (Quote 4). Not openly discussed in the interviews we re-analyzed was the emotional reality of living with someone when these little courtesies can have great importance. The only respondent to do so was Mr Philips (Quote 5), who observes here, that for him, conversation with his wife is now limited to the ‘old times’. Chatting has a special place in the lives of family caregivers Their needs and experiences can be aired and recognized, while conversational etiquette furnishes those living with dementia with opportunities to be addressed and honored as persons [17] and to display—much to a caregivers’ delight—their own, but diminished, social skills. Family caregivers undoubtedly appreciated a ‘chat’; however, this appreciation needs to be seen in conjunction with their more critical estimations of crisis team involvement. Here, this criticism, which has been noted in other studies [18], is set within caregivers’ accounts of caring.

### 3.3. Criticism

Family caregivers were both appreciative and critical of the service they received. Mrs Hillman praised the crisis team for showing ‘empathy, understanding, and support’, but she would have ‘welcomed more [longer]’ involvement from them, while acknowledging that they were ‘obviously stretched for time’. In a similar pairing of praise and critique, Mrs Henderson described the psychiatrist who saw her mother as ‘lovely’ and his assistant as ‘know[ing] his stuff”; yet she is critical of them for not informing her of assessment results (Quote 6). In addition to time-limited involvement and poor communication, concerns were expressed about accessibility. Mr Newman reported feeling ‘reassured’ when he first met the crisis team and was introduced to Suzie. However, Suzie never saw his wife again, and when he ‘desperately needed’ support, his telephone call was not answered (Quote 7). These episodes signal that relational work also has an institutional dimension distinct from the much-appreciated interpersonal realm of chatting. With respect to this institutional dimension, carers are critical of services that fail to meet their expectations but are also willing to excuse those failings. The only carer whose critique spanned both the intuitional and interpersonal dimensions of relational work was Mrs Leyland (Quote 8), who took exception to the idea that the crisis team had solved her problem. These critical comments, it would seem reasonable to assume, are a feature of our interviews, which were designed to elicit views on the qualities of crisis intervention services and are probably not expressed in actual clinical encounters.

In sum, (1) caring is a state of precarious equilibrium; (2) chatting, as relational work, is greatly appreciated by family caregivers, and (3) these caregivers, while critical of the crisis team involvement, are willing to excuse what they perceive as institutional failings.

## 4. Discussion

The findings reported may be of limited generalizability as they draw on data collected from a small sample and for different purposes. Nonetheless, by focusing tightly on what caregivers had to say, the findings reported here notably articulate how the value family caregivers place on relational work as part of the services to support them and the person they care for can stimulate debate and further research into services aimed at supporting people living with dementia to remain longer in their own homes. Two dimensions of relational work can be identified: one, the interpersonal, ‘chatting’, and two, the institutional, which relates to what clients can expect of a service. What is notable is how chatting is greatly appreciated and that in the institutional domain, caregivers are willing to excuse poor service through allusions to the pressures under which health services operate. Specifically, how these two domains interact needs further research: whether and how chatting may influence the willingness of caregivers to continue the difficult work of caring. To the extent that crisis team members can attain the policy goal of sustaining people living with dementia in their own homes, caregivers will still be assigned to continue living within a precarious equilibrium. To the extent that crisis teams fulfill the policy goal of enabling more people to live for longer in their own homes, carrying this out simultaneously assigns greater numbers of caregivers to a precarious equilibrium made vulnerable and stressful by having to constantly respond to a person’s changing and sometimes challenging needs and behaviors. Seeing how caregivers attach such great significance to ‘just’ chatting with a healthcare practitioner indicates how socially isolated [18] and emotionally vulnerable their situation becomes within the care system [19]. This helps explain why Mrs Brown rejects what she characterizes as the crisis team’s attitude that ‘everything is fine now, off we go’ when the social contact she found so valuable was simply removed and not sustained. Recognizing the actions that crisis teams take to reduce inpatient admissions makes apparent that crisis services often do not consider caregivers’ or patients’ relational needs [19], suggesting clear implications for future practice and research.

## 5. Conclusions

There needs to be a more comprehensive understanding of how policies and services designed to enable people with dementia to live for longer in their own homes are and are not impacting family caregivers who bear the brunt of this caring work. Caregivers, as well as patients’ needs, should be seen and addressed in relation to each other and also in terms of the relationality of service practitioners working relationships with them. This more comprehensive relational approach is needed to develop care practices more appropriate to support caregivers and those they care for in the context of their lives in the community. Care practices would therefore need to work so as to recognize the relational as well as clinical needs of caregivers and patients [20] and to better realize the vital role played by family carers [21]. Our findings, based on the observations of caregivers, help specify and understand what substantial role relationality plays in actively enabling all parties involved to engage with each other in resolving crises in dementia care in more dynamic and person-respecting ways. People living with dementia and their caregivers need to be enabled to move beyond assumptions about either the limited relational abilities of people with cognitive disabilities or caregiving roles’ limiting access to relational support for caregivers. Steps to assess these needs should be incorporated into professional clinical practice to build more securely grounded collaborative relationships. Equally important are the relational needs of those providing personal assistance [22], which explicitly contribute to the appreciation of the care approaches that can build up carers’ resilience to difficult long-term care relationships [23]. However, before simply concluding that healthcare practitioners should purposefully devote time to chatting with family caregivers, research would be useful to ascertain how ‘chatting’ with a clinician compares with ‘chatting’ in caregivers support groups [20].

Further and detailed research is therefore needed on the impact of clinicians’ relational work on family caregivers, and specifically, the degree to which and ways in which it influences caregivers’ willingness to carry on caring. To the extent that crisis teams fulfill the policy goal of enabling more people to live for longer in their own homes, this simultaneously assigns greater numbers of caregivers to a precarious equilibrium, as they must respond to challenging needs and behaviors. With respect to challenging behavior in particular, it is important to recognize that such behaviors, if continuing, can place a person at increased risk of inpatient admission, imposing additional stresses on caregivers. Encouraging relationality can contribute to supporting changes in, or at least reducing pressures on, such interactions, recognizing and responding to carers’ needs during a crisis more appropriately than institutionally focused approaches. Better recognizing relational components and contributions to longer-term living would enable practitioners, family caregivers, and the people they care for to identify and more flexibly develop best practices for supporting people with dementia to remain in their homes during and after a crisis.

## Data Availability

The data presented in this study are available on request from the corresponding author due to ongoing data analysis.

## References

[1] Department of Health and Social Care (2009). Living Well with Dementia: A National Dementia Strategy.

[2] Department of Health and Social Care (2015). Prime Minister’s Challenge on Dementia 2020.

[3] Streater A., Coleston-Shields D.M., Yates J., Stanyon M., Orrell M. (2017). A scoping review of crisis teams managing dementia in older people. Clin. Interv. Aging.

[4] Redley M., Poland F., Coleston-Shields D.M., Stanyon M., Yates J., Streater A., Orrell M. (2022). Practitioners’ Views on Enabling People with Dementia to Remain in Their Homes During and After Crisis. J. Appl. Gerontol..

[5] DeFrino D.T. (2009). A theory of the relational work of nurses. Res. Theory Nurs. Pract..

[6] Digby R., Williams A., Lee S. (2016). Nurse empathy and the care of people with dementia. Aust. J. Adv. Nurs..

[7] Broome E.E., Coleston-Shields D.M., Dening T., Moniz-Cook E., Poland F., Stanyon M., Orrell M. (2020). AQUEDUCT Intervention for Crisis Team Quality and Effectiveness in Dementia: Protocol for a Feasibility Study. JMIR Res. Protoc..

[8] Jenks C., Neves T. (2000). A walk on the wild side: Urban ethnography meets the Flâneur. J. Cult. Res..

[9] Lloyd H., Craig S. (2007). A guide to taking a patient’s history. Nurs. Stand..

[10] Silverman D. (2001). Interpreting Qualitative Data: Methods for Analysing Talk, Text and Interaction.

[11] Edwards D., Potter J. (1992). Discursive Psychology.

[12] Mishler E.G. (1991). Research Interviewing: Context and Narrative.

[13] Guest G., MacQueen K.M., Namey E.E. (2011). Applied Thematic Analysis.

[14] Sullivan P. (2012). Qualitative Data Analysis Using a Dialogical Approach Paul Sullivan.

[15] Braun V., Clarke V. (2006). Using thematic analysis in psychology. Qual. Res. Psychol..

[16] Tong A., Sainsbury P., Craig J. (2007). Consolidated criteria for reporting qualitative research (COREQ): A 32-item checklist for interviews and focus groups. Int. J. Qual. Health Care.

[17] Goffman E. (1981). Forms of Talk.

[18] Hopkinson J., King A., Young L., McEwan K., Elliott F., Hydon K., Muthukrishnan S., Tope R., Veitch A.M., Howells C. (2021). Crisis management for people with dementia at home: Mixed-methods case study research to identify critical factors for successful home treatment. Health Soc. Care Community.

[19] Rubinsztein J.S., Hatfield C., High L., Krishnan R., Arnaoutoglou N.A., Goulia P., Dudas R., Ruhi S., Wildschut K., Chouliaras L. (2020). Efficacy of a dementia intensive support (DIS) service at preventing admissions to medical and psychiatric wards: Qualitative and quantitative evaluation. BJPsych Bull..

[20] Ryvicker M., Barrón Y., Shah S., Moore S.M., Noble J.M., Bowles K.H., Merrill J. (2022). Clinical and demographic profiles of home care patients with Alzheimer’s disease and related dementias: Implications for information transfer across care settings. J. Appl. Gerontol..

[21] Griffin J.M., Riffin C., Havyer R.D., Biggar V.S., Comer M., Frangiosa T.L., Bangerter L.R. (2020). Integrating family caregivers of people with Alzheimer’s disease and dementias into clinical appointments: Identifying potential best practices. J. Appl. Gerontol..

[22] Livingston G., Barber J., Rapaport P., Knapp M., Griffin M., King D., Romeo R., Livingston D., Mummery C., Walker Z. (2014). Long-term clinical and cost-effectiveness of psychological intervention for family carers of people with dementia: A single-blind, randomised, controlled trial. Lancet Psychiatry.

[23] Sonola L., Thiel V., Goodwin N., Kodner D. (2013). Oxleas Advanced Dementia Service. Supporting Carers and Building Resilience.

